# Impulse Control Disorders in Parkinson's Disease: Epidemiology, Pathogenesis and Therapeutic Strategies

**DOI:** 10.3389/fpsyt.2021.635494

**Published:** 2021-02-09

**Authors:** Jun-Fang Zhang, Xi-Xi Wang, Ya Feng, Robert Fekete, Joseph Jankovic, Yun-Cheng Wu

**Affiliations:** ^1^Department of Neurology, Shanghai General Hospital, Shanghai Jiao Tong University School of Medicine, Shanghai, China; ^2^Shanghai General Hospital of Nanjing Medical University, Nanjing, China; ^3^Department of Neurology, New York Medical College, New York, NY, United States; ^4^Department of Neurology, Baylor College of Medicine, Houston, TX, United States

**Keywords:** Parkinson's disease, impulse control disorders, dopaminergic drugs, pathological gambling, hypersexuality, binge eating, compulsive buying

## Abstract

Impulse control disorders (ICDs) in Parkinson's disease (PD) are aberrant behavior such as pathological gambling, hypersexuality, binge eating, and compulsive buying, which typically occur as a result of dopaminergic therapy. Numerous studies have focused on the broad spectrum of ICDs-related behaviors and their tremendous impact on patients and their family members. Recent advances have improved our understanding of ICDs. In this review, we discuss the epidemiology, pathogenesis and treatment of ICDs in the setting of PD.

## Highlights

- Impulse control disorders are increasingly recognized as highly impactful features in patients with Parkinson's disease.
- Dopamine receptor agonists are the strongest risk factors.
- The mechanism of impulse control disorders is still not well-understood but the dopamine reward system and inhibition systems are clearly involved.

## Introduction

Parkinson's disease (PD) is the second most common neurodegenerative disorder (1). Chronic use of dopaminergic medications in PD is associated with motor and non-motor side effects such as dyskinesias, and impulse control disorders (ICDs) (2). Motor symptoms of PD have traditionally been the major focus of research, but non-motor symptoms, especially ICDs have gradually attracted great attention because of their tremendous impact on patients and their family (3–5). In general, ICDs refer to pathological gambling (PG), hypersexuality, binge eating, and compulsive buying. The core features of ICDs include repetitive or compulsive behavior, reduced control over these behavior, and pleasurable feeling while carrying out the behavior (4).

PG is defined as persistent and recurrent problematic gambling behavior as indicated by features such as increasing amounts of money, restlessness or irritability when cutting down, failing to control the behavior, preoccupation with gambling, lying to others, and so on (at least four criteria required). However, based on neuropsychiatric and possibly pathophysiological features, PG is currently considered as typical example of behavioral addiction and included in the diagnostic category of “substance-related and addictive disorders” according to DSM-5 (6, 7). Hypersexuality means increasing preoccupation with sexual thought, excessive sexual needs, increased use of pornography and self-stimulatory behavior, seeking out prostitutes, engaging in exhibitionism and paraphilia (8). Binge eating involves uncontrollable consumption of a large amount of food, which results in harmful gain of weight (9). Compulsive buying or shopping can be defined as irresistible excessive buying that can lead to psychological consequences and financial debt (10).

In addition to the ICD, there are also some ICD-related disorders (ICRDs), such as Dopamine dysregulation syndrome (DDS) and punding. DDS implies repeated, unnecessary, or sometimes deleterious daily intake of dopaminergic agents far more than the dosage required for treatment of objective motor impairment, leading to severe dyskinesia, euphoria, aggressivity, hallucination, confusion, or frank psychosis (11). Punding is a term that was coined originally to describe complex prolonged, purposeless, and stereotyped behavior in chronic amphetamine users (12). It shares similarities with addictive behavior and involves psychiatric symptoms relating to dopamine system (13).

Besides the classic ICDs symptoms there are many other ICDs-related behavioral problems including reckless driving (14), impulsive smoking (15), compulsive singing (11), tattooing (16), stealing (17), pet killing (18), and zoophilia (19) (Table 1). The wide clinical spectrum of ICDs symptoms necessitates careful monitoring of behavior when patients are taking dopaminergic drugs. As the researches indicated, dopamine, have been known to have a strong association with ICDs (20).

**Table 1:** Rare ICD symptoms in Parkinson's disease.

**Symptoms**	**Age (yr) and sex**	**Course of PD (yr)**	**Describes**	**Medicine (peak dose)**	**Source**
Reckless driving	65, male; 70, male	9; 20	Impairment in driving performance associated with risk-seeking, including reckless high-speed driving.	L-dopa(-)	(14)
Impulsive smoking	63, male	7	Urge to smoke. Pramipexol was discontinued and the abnormal symptoms disappeared. However, with switching to ropinirole, impulsive smoking developed again.	Pramipexol(6 mg/d)/Ropinirole extended release (12 mg/d)	(15)
Compulsive singing	70, Female; 71, Male	9; 5	Urge to sing repeatedly the same song.	L-dopa(1,268LEU); L-dopa(634 LEU)	(11)
Tattooing	50, Male	–	Got tattooed seven times in 6 months and planned to make five others.	Pramipexol extended release 1.05 mg/d and rasagiline 1 mg/d	(16)
Stealing	48, Female	–	Impulsive stealing.	Pramipexol (-)	(17)
Pet killing	33, Male	–	Compulsive behavior of adopting and killing cats.	Pramipexol (4.5 mg/d)	(18)
Zoophilia	58, Male	20	Attempting to have sexual intercourse with a female family dog.	Pramipexol(8 mg/d)	(19)

*PD indicates parkinson's disease. LEU indicates L-dopa equivalent units*.

This review mainly focuses on the ICDs in PD patients from the point of epidemiology, pathogenesis and therapeutic strategies.

## The Epidemiology of ICDs

The prevalence of ICDs in PD patients using dopamine replacement therapy (DRT) varied from 3.5 to 43% (21–23). Dopamine receptor agonist (DA) treatment in PD is associated with 2–3.5-fold increased odds of having ICDs compared with patients without DA treatment (24). Estimated incidence of ICDs in PD patients increases with time especially in those on DRT (23). In one longitudinal study, the 5-year cumulative incidence of ICDs was about 46% (25). A study showed that 17.5% PD subjects resulted positive with ICDs before starting treatment, indicating the need for a detailed behavioral assessment before dopaminergic therapy (26).

ICD is probably much more frequent in PD than previously reported as patients often underestimate the presence and severity of ICD symptoms (27). This is in part due to lack of insight, but also as a defense mechanism with denial and minimization of symptoms on a background of feelings of shame or guilt. Some patients with ICDs may have a relative lack of empathy and do not perceive any stress from their aberrant behavior, despite marked concerns by family members and friends (28).

## The Risk Factors of ICDs

### Dopaminergic Drugs and ICDs

Although the frequency of impulsivity and compulsive behavior in PD patients before initiation of dopamine receptor agonists is similar to the frequency in healthy control, it is conceivable that dopamine receptor agonists may turn impulsive personality traits into clinically disorders (26). Specifically, affinity of pramipexole and ropinirole for the D3 receptors is much greater than the D2 receptor (100 and 25 times, respectively) as well as for D1 receptor (>1,000 and 300 times, respectively) (29, 30). Other dopamine agonists commercially available in only some countries such as piribedil may also lead to ICDs (31). At the same time, the oral dopamine agonists (pramipexole and ropinirole) have been found to have a greater risk for causing ICDs than the transdermal dopamine agonist rotigotine (29), which might be partially explained by the theory that transdermal delivery bypasses erratic gastric emptying and it may avoid other changes in gastrointestinal motility, leading to the stability of plasma level (29). In a *post-hoc* analysis about PD treated with rotigotine, although no definite conclusion can be reached on any dose-response relationship between rotigotine and ICDs, the incidence of ICDs appeared to increase with the dosage increase (25), as increased with longer exposure to rotigotine and recommend active surveillance with increased duration of treatment and dose reduction when ICDs are present (32).

In addition to dopamine agonists, levodopa, particularly in high dosages, has been also associated with ICDs (24). In patients taking dopamine agonist, concurrent levodopa usage is reported to increase the odds of ICDs by ~50% (24). This multi-center study indicated that there is no association with higher dopamine agonists dose but a link with higher levodopa dose with ICDs, suggesting an intrinsic role for levodopa (33). And also, patients with PD treated by levodopa show ICDs more frequently and more severely than patients without levodopa, thereby suggesting the levodopa's significance in a way (34).

Antidepressants and sleep inducers are also significant predictors for individual ICD (35). Aripiprazole, an antipsychotic drug with partial dopamine agonist properties, has been reported to be associated with ICDs especially pathological gambling (36). It has high affinity for the D3 receptor besides regarded as a D2 agonist. The ICD symptoms resolved completely with its cessation according to reports (36).

In addition to that, studies have failed to find correlation between ICDs and severity of levodopa-related motor fluctuations (37, 38). There were no differences between PD with ICD and PD without ICDs in terms of LID exhibited by DA dose or scores on UPDRS part IV, mania, impulsive choice, alcohol use, or current or former smoking (39).

### Non-Medication Related Risk Factors

#### Demographic Risk Factors

According to current research, many demographic factors participate in the development of ICDs in PD. For example, age, gender, and personality traits. Young age at PD onset is one of most established independent risk factors for ICDs in PD (40). Compared to patients without ICDs, ICDs patients were much younger (21, 24, 25, 29, 41, 42), which can be partly explained by that younger patients are more likely to be prescribed taking dopamine agonists. However, the age effect remains after controlling dopamine agonists exposure (24, 43). Gender difference may contribute to the different subtypes of ICDs. Overall, ICDs increased over time in a more pronounced way in men compared to women (25). Hypersexuality is more prevalent in males while binge eating and buying are more common in women (24, 42, 43). Moreover, male, unmarried, personal or family history of smoking, gambling, drug or alcohol addiction, pre-existent or current symptoms of depression or anxiety and personality traits such as impulsiveness and novelty seeking behaviors are also risk factors of ICDs (4, 37, 41, 42, 44–46). These findings suggest that multiple elements including neurobiological, environmental, genetic factors all contribute to the development of ICDs (24). Furthermore, there are many other personal risk factors, such as depression, anxiety, aggression, irritability, obsessive-compulsive traits, impulsivity traits, novelty seeking traits, and alexithymia (41, 47–50). The inclusion of these factors in the neuropsychiatric assessment of patients with PD may help identify patients at risk for ICDs.

#### Symptomatically Related Risk Factors

The duration of disease or medicine was correlated with ICDs in PD (40). Then the rapid eye movement sleep behavior disorder (RBD) in PD with ICDs is also worth of concern. Actually, whether RBD is a risk factor for ICDs in PD is controversial. Baig et al. (51) recruited 921 cases of PD and screened positive for ICDs at each visit. After statistics, they found that RBD is not associated with increased ICD risk. Another clinical trial involved 401 newly diagnosed PD patients, evaluated ICD behaviors annually and finally revealed that probable RBD is not clearly associated with ICDs in early PD (52). However, a meta-analysis included 10 studies involving 2,781 PD patients drew a conclusion that RBD was associated with a more than 2-fold higher risk of developing ICBs (OR 2.12, *P* < 0.01) (53), reminding us that RBD in PD is confirmed to be a risk factor for impulsive-compulsive behaviors. Hence, more research is needed to explore the role RBD played in PD with ICDs.

### Genetic Risk Factors

There have been studies focusing on DNA polymorphisms of impulsive and compulsive behavior and additive behaviors in decades. A larger number of SNPs in dopaminergic (DRD1 rs 265981, DDC rs 3837091 and rs 1451375, D3Rp.S9G) glutamatergic, serotonergic and opioid neurotransmitter system have been reported as candidates that improved predictability of ICDs when compared with clinical risk factors (54–56). We here cite certain findings that have strong relationship with ICDs. A study indicated that carriage of either AA genotype of DRD3 or CC genotype of GRIN2B was identified as an independent risk factor for ICDs. Furthermore, variants of DRD3 p.S9G and GRIN2B c.366C>G may be associated with ICDs in PD (57). In another study, besides GRIN2B (rs7301328), DRD1 (rs4532 and rs4867798), and DRD2/ANKK1 (rs1800497) increase risk for developing ICDs (58). Polymorphism of DRD4 7-allele also associated with ICDs (59, 60). In addition, a study supported a possible contribution of genetic variation in the HTR2A (serotonin 2A receptor gene) to the susceptibility of ICDs in PD patients, with the T allele, which is presumably linked to higher receptor expression, increasing the risk by 2.8 and 6.9 times in CT and TT carriers (61). More recently, DRD3 p.Ser9Gly (rs6280) CT genotype was proved to be associated with PD patients in Indian population (62). Moreover, with the suggestive association between the opioid receptor gene (OPRM1) and ICDs in PD, the researchers bring potential new insights to the understanding of molecular mechanisms of ICDs (63).

A multicenter case-control study showed that specific subtypes of ICDs, such as compulsive shopping, binge eating and punding, had high frequency and were more severe in PD patients with Parkin mutation compared with non-Parkin mutation (39). The possible explanation was related to neurodegeneration of frontal-striatal-limbic structures (64). In addition, gray matter volume of caudate nuclei, which is involved in reward and stimulus-reinforcement association learning, decreases in PD patients with Parkin mutation (65).

Comparing with single genetic variants, multiple gene interactions may play a more important role. Using candidate genetic multivariable pane, Kraemmer et al. conducted an interesting study to estimate ICD heritability in PD patients, which included several transmitter systems such as dopamine, serotonin, and norepinephrine genes. They found a substantial 11–16% increase in ICD behavior predictability compared to examining clinical variables alone. In addition, in 13 candidate variants, OPRK1, HTR2A and DDC genotypes were the strongest genetic predictive factors and OPRK1 polymorphism rs702764 significantly predicted incident of ICD behavior. Hence, they suggested the potential for developing clinical-genetic models to identify PD patients at increased risk of developing ICD and further guide treatments (66).

Although polymorphisms of dopaminergic genes are not considered as the strongest risk factor for developing ICDs in PD patients at present, further research in the genetic susceptibility will explain the reason why some patients taking low doses of dopaminergic drugs still develop ICDs. Genetic studies can enlarge the understanding of ICDs from pathogenesis to therapy.

## Neuropsychological Studies in ICDs

Various neuropsychological studies have found that ICDs are associated with frontal/executive dysfunctions (67–70). Imbalance of the frontal-striatal circuits which manifested with cognitive dysfunction was considered to be associated with ICDs (71). One study showed that pathological gambling patients performed significantly worse than non-pathological gambling patients in PD on cognitive tasks that evaluated visuo-spatial long-term memory and several frontal lobe functions (68). And PG relates to reward-based decision-making, which is a major topic of behavioral psychology. Clinical neuropsychologists have been using Iowa Gambling Task to evaluate financial risk attitude (72, 73). More straightforward behavioral economics task has been tested. Another study indicated that ICDs patients in PD had poorer working memory performance than either the control or PD patients without ICDs (70). A study found that hypersexuality is associated with prefrontal and memory dysfunctions, whereas pathological gambling and compulsive eating seem to be related to only frontal dysfunction (67).

However, there were several studies considering no difference in frontal executive dysfunction on neuropsychological testing between ICDs and non-ICDs patients in PD (73–78), which suggested that executive dysfunction may contribute to ICD behavior, but is not a necessary component (76). A long-term study investigating the progression of cognitive decline in ICDs patients compared with PD patients without ICDs showed that ICDs patients were not with greater cognitive impairment or executive dysfunction, but rather show relatively lower cognitive decline over time. Drug-induced overstimulation of relatively preserved prefrontal cognitive functions may impair the top-down inhibitory control contributing to ICDs (77). These finding still needs to be verified.

Intertemporal choices, decisions between options available at different times, are commonly applied in impulsivity-related studies. The presence of impulsivity trait in intertemporal choices is usually suggested by a strong preference for small immediate rewards over large delayed ones (79). Temporal discounting is a phenomenon that the subjective valuation of reward declines with delay. In studies using intertemporal choice task, investigators found that dopamine agonist use was associated with greater choice impulsivity in ICDs patients compared to PD. It is suggested that there has been a U-shaped relationship between dopamine activity and temporal discounting (80). Dopamine agonists are associated with a greater discounting of larger delayed rewards, therefore contributing to impulsive choices (69).

## The Pathogenesis of ICDs

**Figure 1:**
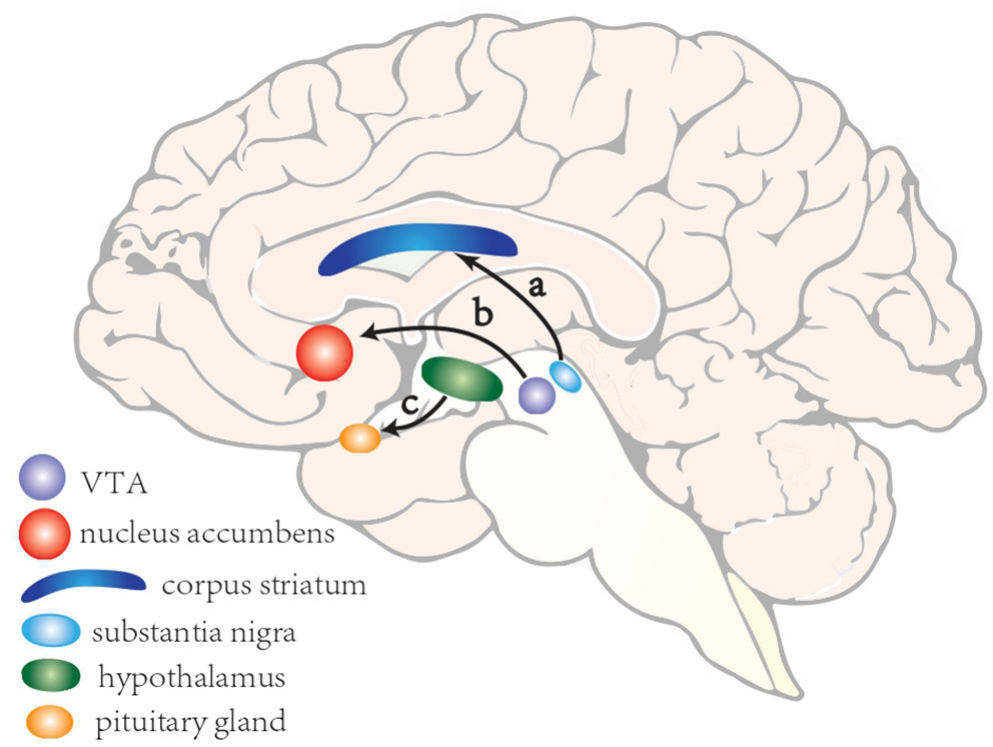
A schematic illustration of three main dopaminergic pathways in central nervous systems. (a) the nigrostriatal pathway consisting of cell bodies in the substantia nigra whose axons terminate in the corpus striatum; (b) the mesocorticolimbic pathway (also known as the reward system), whose cell bodies are situated in the ventral tegmental area and whose axons project to parts of the limbic system; and (c) the tuberoinfundibular pathway, whose cell bodies are found in the ventral hypothalamus and project to the median eminence and pituitary gland.

The mechanism of ICDs is not well-understood. Yet based on animal and clinical researches, the dopaminergic system has been strongly implicated. There are three main dopaminergic pathways in central nervous systems (CNS): (a)the nigrostriatal pathway consisting of cell bodies in the substantia nigra (SN) whose axons terminate in the corpus striatum; (b) the mesocorticolimbic pathway (also known as the reward system), whose cell bodies are situated in the ventral tegmental area and whose axons project to parts of the limbic system; and (c) the tuberoinfundibular pathway, whose cell bodies are found in the ventral hypothalamus and project to the median eminence and pituitary gland (Figure 1). Of the three dopaminergic pathways the mesocorticolimbic system seems to play a key role in the reward system, whose main components include nucleus accumbens, amygdala, hippocampus, anterior cingulate and orbitofrontal cortex (81).

**Figure 2:**
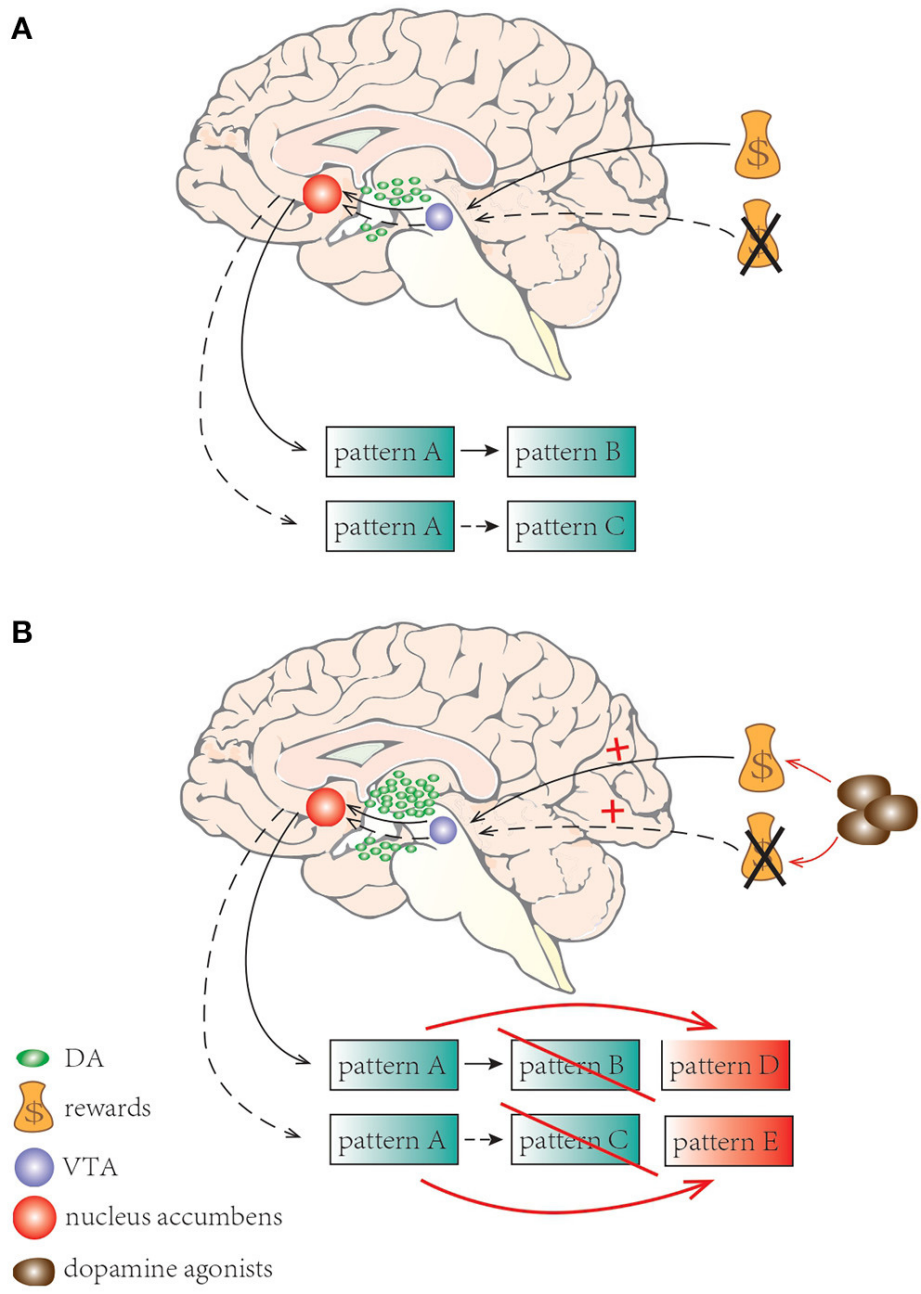
A schematic illustration of learning process and set shifting with dopamine neurotransmission. **(A)** Normally, when expecting a reward or receiving an unexpected reward, phasic release of dopamine from VTA to the nucleus accumbens occurs; phasic suppression of dopamine occurs when an expecting reward is not received. Adaptive behaviors happened shifting from one pattern to a more appropriate one. **(B)** In PD, dopamine agonists may elicit excessive stimulation results in facilitating the appearance of ICDs. ICD patients are less sensitive to negative events but more sensitive to rewarding outcomes.

Dopamine, as a modulator of risk behavior along the mesocorticolimbic pathway, plays an important role in reinforcement of learning. It signals the difference between predicted and experienced reward, and is also involved in shaping behavior to maximize reward and avoid punishment (82). Normally, in anticipation of a reward or when receiving an unexpected reward, phasic release of dopamine from the ventral tegmental area (VTA) to the nucleus accumbens occurs (83). In contrast, phasic suppression of dopamine occurs when an expected reward is not received (83). Contingencies would result in decreasing activation of the mesocorticolimbic dopaminergic system leading to adaptive behavior and shifting from one pattern to a more appropriate action (84) (Figure 2A). In PD, excessive doses of dopamine, dopamine reuptake impairment or stimulation on postsynaptic dopamine receptors by dopaminergic agonists may shift this normal physiologic response and facilitate the appearance of ICDs (57, 82). ICDs patients exhibit reduced ability to learn from negative events and they usually underestimate the adverse consequence of stimuli with punishment, yet they are more sensitive to rewarding outcomes (85) (Figure 2B).

**Figure 3:**
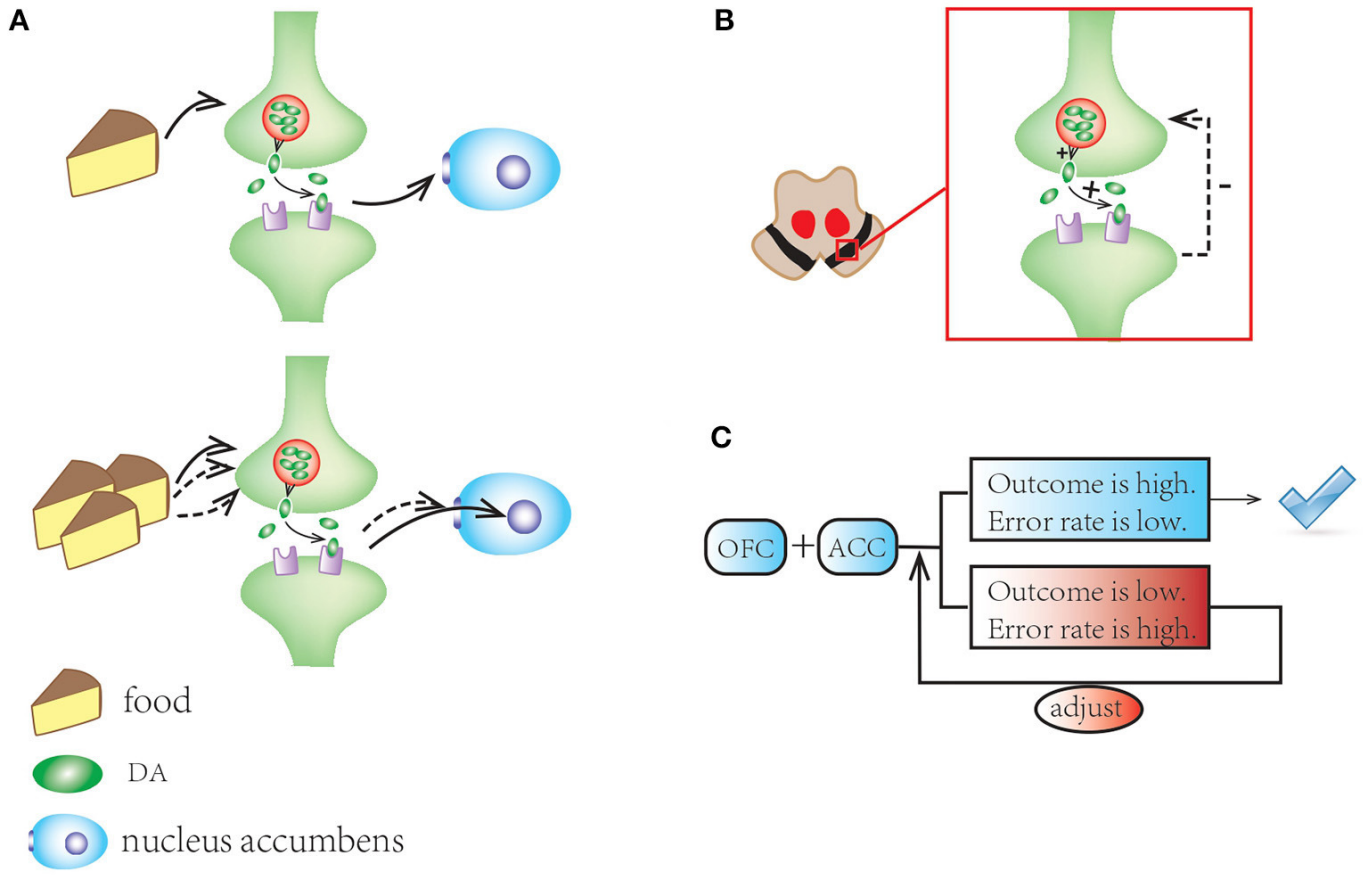
A schematic illustration of mesocorticolimbic network and reward system. **(A)** Standard stimuli (e.g., food) motivate tonic dopamine response in the outer shell of the nucleus accumbens; repeated stimuli induce habitation with the response shifting to its core. **(B)** In substantia nigra, dopamine autoreceptors offer feedback to regulate synaptic dopamine concentrations. **(C)** The OFC and ACC make adjustments for optimizing future choices and balance the system.

There are several observations relevant to reward system and ICDs that are worth highlighting. First, the usual pleasurable stimuli such as food can induce tonic dopamine response in the outer shell of the nucleus accumbens. Second, dopamine auto-receptors in SN provide feedback to regulate synaptic dopamine concentration. Third, the orbitofrontal and anterior cingulate cortices (ACC) are involved in the top-down control, evaluating the reward and directing a suitable reaction, thus making adjustments for optimizing future choices (86). Finally, the prefrontal cortex exerts inhibitory influence to “balance” the system (Figure 3). All these components are working together to help individuals adapt to their environment.

PD is associated with a neurodegenerative process that involves mesocorticolimbic network, but there is paucity of data linking abnormalities of this network to ICDs. One study, using volumetric magnetic resonance imaging (MRI) technique, found that amygdala volume was greater in PD patients with ICDs than those without ICDs, but similar to health control subjects (64). Since amygdala is important in processing both positive and aversive emotional inputs, relatively preserved amygdala is needed for the expression of ICDs (87, 88). Another study showed that ICDs patients have a thicker cortex in certain limbic regions especially in ACC and orbitofrontal cortex, which may be linked to increased impulsivity and behavioral disinhibition (89). In addition, this study demonstrated positive correlation between the ACC thickening and ICD severity (89). Moreover, a whole-brain diffusion-tensor MRI study found white-matter integrity in the reward system is relatively preserved in ICDs-PD patients compared to PD patients without ICDs (90). A BOLD fMRI study observed that PD patients with ICDs had elevated network connectivity in the mesocorticolimbic network (91).

The mesocorticolimbic reward system plays an important role in the development and maintenance of addictive behavior. The reason for male preponderance in patients exhibiting this behavior is still unclear, but some imaging studies have suggested that males have a stronger functional connectivity in the reward mesocorticolimbic system than females (29, 92). Thus, the relative preservation of neural integrity in mesocorticolimbic network and the intact reward-processing circuits are thought to increase risk for ICDs in PD patients treated with dopaminergic medications (90).

In addition to the reward system, ICDs are also involved in the inhibition system. One fMRI study showed impairment in response-inhibition abilities in ICDs patients (93). The observation found that the rostral portion of the corpus callosum in ICDs patients is thinner compared to healthy control. And it has been interpreted to indicate that there might be some disconnection in the inhibitory system normally mediated by the corpus callosum, leading to behavioral disinhibition and ICDs. The normal inhibitory mechanisms may be further disrupted by treatment with dopaminergic drugs which may explain why some PD patients become vulnerable and experience loss of impulse control (disinhibition) and finally results in the development of ICDs (94). In support of this hypothesis is the finding that pramipexole decreases the interaction between the nucleus accumbens and prefrontal cortex, which might lead to a reduction of normal prefrontal inhibitory control of impulses (95). Thus, dopamine agonists presumably act by suppressing the inhibitory system and elicit a response bias toward impulsive choices (96).

Dopamine agonists improve motor symptoms in PD patients through their effects on the dorsal striatum, but they also activate the ventral striatum and the mesolimbic pathway (97). The brain activity in ventral striatum can be separated spatially and temporally into signals correlated with risk and reward expectation which are both the foundation of decision-making (98). In addition to the involvement of the striatum in ICDs, several studies have implicated the ventral pallidum in the modulation of hedonic responses to rewards (99). Using arterial-spin-labeling MRI, increased cerebral blood flow to ventral striatum in ICDs patients had been substantiated in response to dopamine agonists, which indicated that dopamine agonists can augment mesocorticolimbic network activity in ICDs patients (100). The function of the orbitofrontal cortex (OFC) is critical in understanding the mechanisms of rewards or punishments (65, 101). The OFC may be activated by stimuli from reward-related memories or environment which then induces a strong sense of urge with or without activation of the nucleus accumbens (102). While the medial OFC engages in reward-based decision-making the lateral OFC is associated with the punishment-based decision-making (101). The role of OFC in ICDs is supported by studies that have found dysfunction of impaired long-term memory and frontal lobe functions in patients exhibiting pathological gambling compared to control (68). Other studies have found evidence that frontal lobe dysfunction facilitates the onset and persistence of pathological gambling in PD (103).

## Therapeutic Strategies for ICDs

In ICDs patients with PD, caregivers suffer huge burden from mental stress specifically on spousal safety. ICDs are associated with high rate of separation and divorce, child abuse, and neglect (4, 104, 105). It is important to recognize the disease and treat it without delay.

As discussed above, ICDs are considered to have strong relationship with dopamine agonists. Decreasing or even withdrawing dopamine agonists is usually the first choice for clinicians. For PD patients who have developed ICDs on account of dopamine agonist treatment, they will get remission significantly after decreasing dosages. One longitudinal study suggested that ICDs resolved after 1 year in about 50% of the patients who stopped dopamine agonists and continued to improve (25). Patients can increase levodopa dosage instead to avoid worsening in motor symptoms (106). However, treatment is still challenging, as patients may experience dopamine agonist withdrawal syndrome (107–109).

Considering the benefit from reducing dose of dopamine agonists such as pramipexole or ropinirole, it is recommended that temporary replacement of pramipexole by bromocriptine instead may relieve or reverse the ICDs while the D2 stimulation needed for motor symptoms are still maintained (95).

Subthalamic nucleus (STN) deep brain stimulation (DBS) as a treatment for ICDs is considered a controversial method according to reported literature. Compared to patients without ICDs, ICDs patients exhibited increased proportion of subthalamic neurons responsive to prospective reward and decreased proportion to prospective loss in STN but no in GPi (110). STN-DBS and the following tapering of dopaminergic treatment can change personality traits in PD patients (111). A few studies are in favor of DBS surgery as a treatment for ICDs and even suggest that ICDs may be considered as new indication for STN-DBS (112, 113). ICDs patients exhibited a complex outcome after STN-DBS, with a tendency for overall reduction but with several factors affecting its effect (114). It is believed that effective management of medication and correct stimulation parameters may explain these results better than previous literature. Successful surgery allows a marked decrease of total dopaminergic medication. The STN stimulation may also have specific effect on limbic part of the STN (115). In general, fine-tuning of stimulation parameter after DBS surgery, accompanied with drastic reduction of dopaminergic medication are considered as an effective method to give remission to ICDs patients especially advanced subgroup (116, 117). Furthermore, there has been a study indicating that unilateral procedures may be an alternative to bilateral DBS for some patients if they are with asymmetric symptomology (118). Meanwhile, some studies disapprove of using STN-DBS because ICDs may persist or even worsen after DBS surgery (119). Even more, some evidence shows that ICDs may emerge following DBS surgery regardless of unilateral or bilateral DBS (120–122). Stimulation by DBS might sensitize the brain to the impulsive behaviors induced by dopamine agonists, especially in patients with addictive behavior history (121). Besides, stimulation with electrode contacts located mainly within the sensorimotor territory can result in spread of current to limbic and associative area (123). Failed surgery, with misplaced electrodes outside the STN, would result in failure to reduce dopaminergic medication or even causing new onset of dopaminergic treatment (112). Stimulation intensity increased too rapidly will elicit ICDs in the same way as dopaminergic treatment (124). Therefore, we should be careful when choosing DBS treatment in clinical practices.

Amantadine, acting as a dopaminergic and glutamatergic modulator, was reported to have great effect on reversing ICD symptoms without aggravating motor function. Amantadine add-on therapy is considered to reduce hypersensitivity in ICDs patients therefore decrease risky choice (125–127). However, amantadine was associated with an increased risk for ICDs in another multicenter study (128).

As ICDs are thought to be linked to oral dopamine agonists, strategies utilizing intrajejunal levodopa which utilize continuous drug delivery may decrease the risk of developing ICDs. This therapy may become a popular treatment of ICDs not only because its positive effect on behavioral disorders but also motor complications (129, 130).

Clozapine were reported as a potential treatment for refractory ICDs. Clozapine not only has an effect on dopamine-blocking activity in the limbic system but also has weak antagonistic D3 and high antagonistic D4 activity that makes it capable of adjusting the reward circuit. Besides, N-desmethyloclozapine, the major active plasma metabolite of clozapine, may have an important partial agonist activity on dopamine D2/D3 receptors (131). There have been several cases reporting beneficial responses to clozapine in ICDs patients (132, 133).

There has been an emerging method to treat ICDs using transcranial magnetic stimulation (TMS). A study reported that low-frequency repetitive TMS was used to treat four PD patients with punding whose symptoms were reversed magically. TMS deserves more studies to explore the best pattern and more indications (134).

To date, there have been few studies concerning the role of cognitive behavior therapy in ICDs. Okai et al. proved the efficacy of cognitive behavior treatment in ICD patients with PD through a randomized controlled trail. They found the combined treatment of cognitive behavior treatment with medical care was more effective in reducing the severity of ICDs compared with medical care alone. The severity of symptom (measured on the Clinical Global Impression (CGI) index) significantly reduced 75% of the experimental group compared with only 29% in the control group. The intervention seemed to be also effective in depression and anxiety. Larger and long-term follow up studies are needed to confirm the benefit of cognitive behavior treatment in each subtype of ICDs, and meanwhile assess the cost-effectiveness (135).

In addition to those treatments discussed above, valproate, zonisamide, naloxone, apomorphine, and bromocriptine may also be beneficial in treating ICDs (136, 137). Dopamine agonists with lower D3 selectivity appear to have lower proportion of causing ICDs. Switching to bromocriptine was proposed as a method to mitigate ICDs. More research and clinical trials are needed to explore the best therapeutic strategy.

## Conclusion

In conclusion, present studies remind us to pay much attention to non-motor symptoms including ICDs. With rapid advance regarding to ICDs, mechanisms of ICDs will become gradually clear and specific individual treatment strategies will be applied in the future. Given that ICDs would have terrible impact and consequence on families, patients and their caregivers should be educated in clinical practice. In addition, ICDs in PD patients may also provide a model for better understanding of the neurobiology of addiction.

## Author Contributions

Y-CW provided the funding support and designed the project. J-FZ, X-XW, and YF searched for literature and wrote the manuscript. Y-CW, RF, and JJ revised the paper. All authors contributed to the article and approved the submitted version.

## Conflict of Interest

The authors declare that the research was conducted in the absence of any commercial or financial relationships that could be construed as a potential conflict of interest.

## Abbreviations

ICDs: Impulse control disorders
PD: Parkinson's disease
PG: Pathological gambling
DDS: Dopamine dysregulation syndrome
DRT: Dopamine replacement therapy
DAs: Dopamine agonists
ACC: anterior cingulate cortices
OFC: orbitofrontal cortex
VTA: ventral tegmental area
DAT: Dopamine transporter
RBD: Rapid eyes movement sleep behavior disorder
STN: Subthalamic nucleus
DBS: Deep brain stimulation
TMS: Transcranial magnetic stimulation.

## References

[1] de Lau LM Breteler MM Epidemiology of Parkinson's disease. Lancet Neurol. (2006) 5:525–35. 10.1016/S1474-4422(06)70471-9 16713924

[2] Voon V Napier TC Frank MJ Sgambato-Faure V Grace AA Rodriguez-Oroz M. Impulse control disorders and levodopa-induced dyskinesias in Parkinson's disease: an update. Lancet Neurol. (2017) 16:238–50. 10.1016/S1474-4422(17)30004-2 28229895

[3] Weintraub D Mamikonyan E Impulse control disorders in Parkinson's disease. Am J Psychiatry. (2019) 176:5–11. 10.1176/appi.ajp.2018.18040465 30848949

[4] Ceravolo R Frosini D Rossi C Bonuccelli U. Impulse control disorders in Parkinson's disease: definition, epidemiology, risk factors, neurobiology and management. Parkinsonism Relat Disord. (2009) 15(Suppl. 4):S111–5. 10.1016/S1353-8020(09)70847-8 20123548

[5] Moegle C Grillon A Anheim M Lipsker D Velter C. Impulse control disorder-linked hypersexuality complicated by disseminated gonococcal infection in a patient with Parkinson's disease. Rev Neurol. (2020) 176:292–3. 10.1016/j.neurol.2019.10.007 32139181

[6] Nautiyal KM Okuda M Hen R Blanco C. Gambling disorder: an integrative review of animal and human studies. Ann N Y Acad Sci. (2017) 1394:106–27. 10.1111/nyas.13356 28486792 PMC5466885

[7] Goudriaan AE Yucel M van Holst RJ. Getting a grip on problem gambling: what can neuroscience tell us? Front Behav Neurosci. (2014) 8:141. 10.3389/fnbeh.2014.00141 24904328 PMC4033022

[8] Lim SY Evans AH Miyasaki JM. Impulse control and related disorders in Parkinson's disease: review. Ann N Y Acad Sci. (2008) 1142:85–107. 10.1196/annals.1444.006 18990123

[9] Nirenberg MJ Waters C. Compulsive eating and weight gain related to dopamine agonist use. Mov Disord. (2006) 21:524–9. 10.1002/mds.20757 16261618

[10] Dittmar H. Compulsive buying–a growing concern? An examination of gender, age, and endorsement of materialistic values as predictors. Br J Psychol. (2005) 96:467–91. 10.1348/000712605X53533 16248937

[11] Bonvin C Horvath J Christe B Landis T Burkhard PR. Compulsive singing: another aspect of punding in Parkinson's disease. Ann Neurol. (2007) 62:525–8. 10.1002/ana.21202 17696122

[12] Evans AH Katzenschlager R Paviour D O'Sullivan JD Appel S Lawrence AD. Punding in Parkinson's disease: its relation to the dopamine dysregulation syndrome. Mov Disord. (2004) 19:397–405. 10.1002/mds.20045 15077237

[13] Pettorruso M Fasano A De Risio L Ricciardi L Di Nicola M Martinotti G. Punding in non-demented Parkinson's disease patients: relationship with psychiatric and addiction spectrum comorbidity. J Neurol Sci. (2016) 362:344–7. 10.1016/j.jns.2016.02.016 26944176

[14] Avanzi M Baratti M Cabrini S Uber E Brighetti G Bonfa F. The thrill of reckless driving in patients with Parkinson's disease: an additional behavioural phenomenon in dopamine dysregulation syndrome? Parkinsonism Relat Disord. (2008) 14:257–8. 10.1016/j.parkreldis.2007.04.006 17561433

[15] Bienfait KL Menza M Mark MH Dobkin RD. Impulsive smoking in a patient with Parkinson's disease treated with dopamine agonists. J Clin Neurosci. (2010) 17:539–40. 10.1016/j.jocn.2009.09.001 20171891 PMC2834808

[16] Maltete D Le Goff F Ozel G Lefaucheur R. Tattooing as a symptom of impulse control disorder in a parkinsonian patient with pramipexole. J Clin Psychopharmacol. (2016) 36:736–7. 10.1097/JCP.0000000000000585 27749676

[17] Clemm von Hohenberg C Dressing H. Stealing as an impulse control disorder associated with pramipexole - a case report from forensic psychiatric practice. Psychiatr Prax. (2017) 44:172–4. 10.1055/s-0043-100024 28399600

[18] Micheli F Pellene A Arcushin D Calzinari A Farret MS. Pet killing as a manifestation of impulse control disorder secondary to pramipexol. Clin Neuropharmacol. (2015) 38:55–6. 10.1097/WNF.0000000000000074 25768852

[19] Raina G Cersosimo MG Micheli F. Zoophilia and impulse control disorder in a patient with Parkinson disease. J Neurol. (2012) 259:969–70. 10.1007/s00415-011-6270-z 22057400

[20] Moore TJ Glenmullen J Mattison DR. Reports of pathological gambling, hypersexuality, and compulsive shopping associated with dopamine receptor agonist drugs. JAMA Intern Med. (2014) 174:1930–3. 10.1001/jamainternmed.2014.5262 25329919

[21] Ramirez Gomez CC Serrano Duenas M Bernal O Araoz N Saenz Farret M Aldinio V. A multicenter comparative study of impulse control disorder in latin American patients with Parkinson disease. Clin Neuropharmacol. (2017) 40:51–5. 10.1097/WNF.0000000000000202 28288482

[22] Ambermoon P Carter A Hall WD Dissanayaka NN O'Sullivan JD. Impulse control disorders in patients with Parkinson's disease receiving dopamine replacement therapy: evidence and implications for the addictions field. Addiction. (2011) 106:283–93. 10.1111/j.1360-0443.2010.03218.x 21134016

[23] Smith KM Xie SX Weintraub D. Incident impulse control disorder symptoms and dopamine transporter imaging in Parkinson disease. J Neurol Neurosurg Psychiatry. (2016) 87:864–70. 10.1136/jnnp-2015-311827 26534930 PMC4854795

[24] Weintraub D Koester J Potenza MN Siderowf AD Stacy M Voon V. Impulse control disorders in Parkinson disease: a cross-sectional study of 3090 patients. Arch Neurol. (2010) 67:589–95. 10.1001/archneurol.2010.65 20457959

[25] Corvol JC Artaud F Cormier-Dequaire F Rascol O Durif F Derkinderen P. Longitudinal analysis of impulse control disorders in Parkinson disease. Neurology. (2018) 91:e189–201. 10.1212/WNL.0000000000005816 29925549 PMC6059034

[26] Antonini A Siri C Santangelo G Cilia R Poletti M Canesi M. Impulsivity and compulsivity in drug-naive patients with Parkinson's disease. Mov Disord. (2011) 26:464–8. 10.1002/mds.23501 21312278

[27] Baumann-Vogel H Valko PO Eisele G Baumann CR. Impulse control disorders in Parkinson's disease: don't set your mind at rest by self-assessments. Eur J Neurol. (2015) 22:603–9. 10.1111/ene.12646 25598147

[28] Tomei A Besson J Grivel J. Linking empathy to visuospatial perspective-taking in gambling addiction. Psychiatry Res. (2017) 250:177–84. 10.1016/j.psychres.2016.12.061 28161613

[29] Garcia-Ruiz PJ Martinez Castrillo JC Alonso-Canovas A Herranz Barcenas A Vela L Sanchez Alonso P. Impulse control disorder in patients with Parkinson's disease under dopamine agonist therapy: a multicentre study. J Neurol Neurosurg Psychiatry. (2014) 85:840–4. 10.1136/jnnp-2013-306787 24434037

[30] Martinez-Castrillo JC. Impulse control disorders in Parkinson's disease: a hard-turning point. J Neurol Neurosurg Psychiatry. (2019) 90:2. 10.1136/jnnp-2018-319375 30361297

[31] Micheli FE Giugni JC Espinosa ME Calvo DS Raina GB. Piribedil and pathological gambling in six parkinsonian patients. Arq Neuropsiquiatr. (2015) 73:115–8. 10.1590/0004-282X20140212 25742580

[32] Antonini A Chaudhuri KR Boroojerdi B Asgharnejad M Bauer L Grieger F. Impulse control disorder related behaviours during long-term rotigotine treatment: a post hoc analysis. Eur J Neurol. (2016) 23:1556–65. 10.1111/ene.13078 27425586 PMC5096013

[33] Voon V Fernagut PO Wickens J Baunez C Rodriguez M Pavon N. Chronic dopaminergic stimulation in Parkinson's disease: from dyskinesias to impulse control disorders. Lancet Neurol. (2009) 8:1140–9. 10.1016/S1474-4422(09)70287-X 19909912

[34] Simoni S Paoletti FP Eusebi P Cappelletti G Filidei M Brahimi E. Impulse control disorders and levodopa-induced dyskinesias in Parkinson's disease: pulsatile versus continuous dopaminergic stimulation. J Parkinsons Dis. (2020) 10:927–34. 10.3233/JPD-191833 32280105

[35] Carbunaru S Eisinger RS Ramirez-Zamora A Bassan D Cervantes-Arriaga A Rodriguez-Violante M. Impulse control disorders in Parkinson's: sleep disorders and nondopaminergic associations. Brain Behav. (2018) 8:e00904. 10.1002/brb3.904 29541533 PMC5840436

[36] Dhillon R Bastiampillai T Cao CZ Eckert TG Tibrewal P. Aripiprazole and impulse-control disorders: a recent FDA warning and a case report. Prim Care Companion CNS Disord. (2017) 19:1. 10.4088/PCC.16l02003 28102975

[37] Antonini A Barone P Bonuccelli U Annoni K Asgharnejad M Stanzione P. ICARUS study: prevalence and clinical features of impulse control disorders in Parkinson's disease. J Neurol Neurosurg Psychiatry. (2017) 88:317–24. 10.1136/jnnp-2016-315277 28315845

[38] Storch A Schneider CB Wolz M Sturwald Y Nebe A Odin P. Nonmotor fluctuations in Parkinson disease: severity and correlation with motor complications. Neurology. (2013) 80:800–9. 10.1212/WNL.0b013e318285c0ed 23365054

[39] Morgante F Fasano A Ginevrino M Petrucci S Ricciardi L Bove F. Impulsive-compulsive behaviors in parkin-associated Parkinson disease. Neurology. (2016) 87:1436–41. 10.1212/WNL.0000000000003177 27590295 PMC5075971

[40] Giladi N Weitzman N Schreiber S Shabtai H Peretz C. New onset heightened interest or drive for gambling, shopping, eating or sexual activity in patients with Parkinson's disease: the role of dopamine agonist treatment and age at motor symptoms onset. J Psychopharmacol. (2007) 21:501–6. 10.1177/0269881106073109 17446202

[41] Voon V Sohr M Lang AE Potenza MN Siderowf AD Whetteckey J. Impulse control disorders in Parkinson disease: a multicenter case–control study. Ann Neurol. (2011) 69:986–96. 10.1002/ana.22356 21416496

[42] Vela L Martinez Castrillo JC Garcia Ruiz P Gasca-Salas C Macias Macias Y Perez Fernandez E. The high prevalence of impulse control behaviors in patients with early-onset Parkinson's disease: a cross-sectional multicenter study. J Neurol Sci. (2016) 368:150–4. 10.1016/j.jns.2016.07.003 27538621

[43] Nakum S Cavanna AE. The prevalence and clinical characteristics of hypersexuality in patients with Parkinson's disease following dopaminergic therapy: a systematic literature review. Parkinsonism Relat Disord. (2016) 25:10–6. 10.1016/j.parkreldis.2016.02.017 26923525

[44] Wolters E van der Werf YD van den Heuvel OA. Parkinson's disease-related disorders in the impulsive-compulsive spectrum. J Neurol. (2008) 255(Suppl. 5):48–56. 10.1007/s00415-008-5010-5 18787882

[45] Saez-Francas N Marti Andres G Ramirez N de Fabregues O Alvarez-Sabin J Casas M. Clinical and psychopathological factors associated with impulse control disorders in Parkinson's disease. Neurologia. (2016) 31:231–8. 10.1016/j.nrleng.2015.05.008 26096669

[46] Martini A Dal Lago D Edelstyn NMJ Grange JA Tamburin S. Impulse control disorder in Parkinson's disease: a meta-analysis of cognitive, affective, and motivational correlates. Front Neurol. (2018) 9:654. 10.3389/fneur.2018.00654 30233478 PMC6127647

[47] Goerlich-Dobre KS Probst C Winter L Witt K Deuschl G Moller B. Alexithymia-an independent risk factor for impulsive-compulsive disorders in Parkinson's disease. Mov Disord. (2014) 29:214–20. 10.1002/mds.25679 24123483

[48] Pontone G Williams JR Bassett SS Marsh L. Clinical features associated with impulse control disorders in Parkinson disease. Neurology. (2006) 67:1258–61. 10.1212/01.wnl.0000238401.76928.45 17030761

[49] Voon V Thomsen T Miyasaki JM de Souza M Shafro A Fox SH. Factors associated with dopaminergic drug-related pathological gambling in Parkinson disease. Arch Neurol. (2007) 64:212–6. 10.1001/archneur.64.2.212 17296836

[50] Isaias IU Siri C Cilia R De Gaspari D Pezzoli G Antonini A. The relationship between impulsivity and impulse control disorders in Parkinson's disease. Mov Disord. (2008) 23:411–5. 10.1002/mds.21872 18067187

[51] Baig F Kelly MJ Lawton MA Ruffmann C Rolinski M Klein JC. Impulse control disorders in Parkinson disease and RBD: a longitudinal study of severity. Neurology. (2019) 93:e675–87. 10.1212/WNL.0000000000007942 31311842 PMC6715510

[52] Fantini ML Fedler J Pereira B Weintraub D Marques AR Durif F. Is rapid eye movement sleep behavior disorder a risk factor for impulse control disorder in Parkinson disease? Ann Neurol. (2020) 88:759–70. 10.1002/ana.25798 32468593

[53] Lu HT Shen QY Zhao QZ Huang HY Ning PP Wang H. Association between REM sleep behavior disorder and impulsive-compulsive behaviors in Parkinson's disease: a systematic review and meta-analysis of observational studies. J Neurol. (2020) 267:331–40. 10.1007/s00415-019-09588-3 31637489

[54] Jimenez-Urbieta H Gago B de la Riva P Delgado-Alvarado M Marin C Rodriguez-Oroz MC. Dyskinesias and impulse control disorders in Parkinson's disease: from pathogenesis to potential therapeutic approaches. Neurosci Biobehav Rev. (2015) 56:294–314. 10.1016/j.neubiorev.2015.07.010 26216865

[55] Weintraub D Claassen DO Impulse control and related disorders in Parkinson's disease. Int Rev Neurobiol. (2017) 133:679–717. 10.1016/bs.irn.2017.04.006 28802938

[56] Bhattacharjee S Talbot JG Vijayashankar P. Dopamine D3 receptor Ser9Gly variant is associated with impulse control disorders in Parkinson's disease patients. Parkinsonism Relat Disord. (2017) 34:69–70. 10.1016/j.parkreldis.2016.10.021 27802909

[57] Lee JY Lee EK Park SS Lim JY Kim HJ Kim JS. Association of DRD3 and GRIN2B with impulse control and related behaviors in Parkinson's disease. Mov Disord. (2009) 24:1803–10. 10.1002/mds.22678 19562769

[58] Zainal Abidin S Tan EL Chan SC Jaafar A Lee AX Abd Hamid MH. DRD and GRIN2B polymorphisms and their association with the development of impulse control behaviour among Malaysian Parkinson's disease patients. BMC Neurol. (2015) 15:59. 10.1186/s12883-015-0316-2 25896831 PMC4417293

[59] Comings DE Gonzalez N Wu S Gade R Muhleman D Saucier G. Studies of the 48 bp repeat polymorphism of the DRD4 gene in impulsive, compulsive, addictive behaviors: Tourette syndrome, ADHD, pathological gambling, and substance abuse. Am J Med Genet. (1999) 88:358–68. 10402503 10.1002/(sici)1096-8628(19990820)88:4<358::aid-ajmg13>3.0.co;2-g

[60] Forbes EE Brown SM Kimak M Ferrell RE Manuck SB Hariri AR. Genetic variation in components of dopamine neurotransmission impacts ventral striatal reactivity associated with impulsivity. Mol Psychiatry. (2009) 14:60–70. 10.1038/sj.mp.4002086 17893706 PMC2668513

[61] Lee JY Jeon BS Kim HJ Park SS. Genetic variant of HTR2A associates with risk of impulse control and repetitive behaviors in Parkinson's disease. Parkinsonism Relat Disord. (2012) 18:76–8. 10.1016/j.parkreldis.2011.08.009 21900033

[62] Krishnamoorthy S Rajan R Banerjee M Kumar H Sarma G Krishnan S. Dopamine D3 receptor Ser9Gly variant is associated with impulse control disorders in Parkinson's disease patients. Parkinsonism Relat Disord. (2016) 30:13–7. 10.1016/j.parkreldis.2016.06.005 27325396

[63] Cormier-Dequaire F Bekadar S Anheim M Lebbah S Pelissolo A Krack P. Suggestive association between OPRM1 and impulse control disorders in Parkinson's disease. Mov Disord. (2018) 33:1878–86. 10.1002/mds.27519 30444952

[64] Biundo R Weis L Facchini S Formento-Dojot P Vallelunga A Pilleri M. Patterns of cortical thickness associated with impulse control disorders in Parkinson's disease. Mov Disord. (2015) 30:688–95. 10.1002/mds.26154 25649923

[65] Rolls ET The orbitofrontal cortex and reward. Cereb Cortex. (2000) 10:284–94. 10.1093/cercor/10.3.284 10731223

[66] Kraemmer J Smith K Weintraub D Guillemot V Nalls MA Cormier-Dequaire F. Clinical-genetic model predicts incident impulse control disorders in Parkinson's disease. J Neurol Neurosurg Psychiatry. (2016) 87:1106–111. 10.1136/jnnp-2015-312848 27076492 PMC5098340

[67] Vitale C Santangelo G Trojano L Verde F Rocco M Grossi D. Comparative neuropsychological profile of pathological gambling, hypersexuality, and compulsive eating in Parkinson's disease. Mov Disord. (2011) 26:830–836. 10.1002/mds.23567 21370268

[68] Santangelo G Vitale C Trojano L Verde F Grossi D Barone P. Cognitive dysfunctions and pathological gambling in patients with Parkinson's disease. Mov Disord. (2009) 24:899–905. 10.1002/mds.22472 19205072

[69] Voon V Reynolds B Brezing C Gallea C Skaljic M Ekanayake V. Impulsive choice and response in dopamine agonist-related impulse control behaviors. Psychopharmacology. (2010) 207:645–59. 10.1007/s00213-009-1697-y 19838863 PMC3676926

[70] Djamshidian A Jha A O'Sullivan SS Silveira-Moriyama L Jacobson C Brown P. Risk and learning in impulsive and nonimpulsive patients with Parkinson's disease. Mov Disord. (2010) 25:2203–10. 10.1002/mds.23247 20721918 PMC3093055

[71] van den Heuvel OA van der Werf YD Verhoef KM de Wit S Berendse HW Wolters E. Frontal-striatal abnormalities underlying behaviours in the compulsive-impulsive spectrum. J Neurol Sci. (2010) 289:55–9. 10.1016/j.jns.2009.08.043 19729172

[72] Kobayakawa M Tsuruya N Kawamura M. Sensitivity to reward and punishment in Parkinson's disease: an analysis of behavioral patterns using a modified version of the Iowa gambling task. Parkinsonism Relat Disord. (2010) 16:453–7. 10.1016/j.parkreldis.2010.04.011 20493754

[73] Rossi M Gerschcovich ER de Achaval D Perez-Lloret S Cerquetti D Cammarota A. Decision-making in Parkinson's disease patients with and without pathological gambling. Eur J Neurol. (2010) 17:97–102. 10.1111/j.1468-1331.2009.02792.x 19780806

[74] Pineau F Roze E Lacomblez L Bonnet AM Vidailhet M Czernecki V. Executive functioning and risk-taking behavior in Parkinson's disease patients with impulse control disorders. J Neural Transm. (2016) 123:573–81. 10.1007/s00702-016-1549-y 27085342

[75] Siri C Cilia R De Gaspari D Canesi M Meucci N Zecchinelli AL. Cognitive status of patients with Parkinson's disease and pathological gambling. J Neurol. (2010) 257:247–52. 10.1007/s00415-009-5301-5 19727901

[76] Mack J Okai D Brown RG Askey-Jones S Chaudhuri KR Martin A. The role of self-awareness and cognitive dysfunction in Parkinson's disease with and without impulse-control disorder. J Neuropsychiatry Clin Neurosci. (2013) 25:141–9. 10.1176/appi.neuropsych.12030076 23686032

[77] Siri C Cilia R Reali E Pozzi B Cereda E Colombo A. Long-term cognitive follow-up of Parkinson's disease patients with impulse control disorders. Mov Disord. (2015) 30:696–704. 10.1002/mds.26160 25757654

[78] Pontieri FE Assogna F Pellicano C Cacciari C Pannunzi S Morrone A. Sociodemographic, neuropsychiatric and cognitive characteristics of pathological gambling and impulse control disorders NOS in Parkinson's disease. Eur Neuropsychopharmacol. (2015) 25:69–76. 10.1016/j.euroneuro.2014.11.006 25435085

[79] Milenkova M Mohammadi B Kollewe K Schrader C Fellbrich A Wittfoth M Intertemporal choice in Parkinson's disease. Mov Disord. (2011) 26:2004–10. 10.1002/mds.23756 21567457

[80] Joutsa J Voon V Johansson J Niemela S Bergman J Kaasinen V Dopaminergic function and intertemporal choice. Transl Psychiatry. (2015) 5:e491 10.1038/tp.2014.133 25562841 PMC4312827

[81] Aracil-Bolanos I Strafella AP. Molecular imaging and neural networks in impulse control disorders in Parkinson's disease. Parkinsonism Relat Disord. (2016) 22(Suppl. 1):S101–5. 10.1016/j.parkreldis.2015.08.003 26298389 PMC4874782

[82] Clark CA Dagher A. The role of dopamine in risk taking: a specific look at Parkinson's disease and gambling. Front Behav Neurosci. (2014) 8:196. 10.3389/fnbeh.2014.00196 24910600 PMC4038955

[83] Schultz W Dayan P Montague PR. A neural substrate of prediction and reward. Science. (1997) 275:1593–9. 10.1126/science.275.5306.1593 9054347

[84] Ferron A Thierry AM Le Douarin C Glowinski J. Inhibitory influence of the mesocortical dopaminergic system on spontaneous activity or excitatory response induced from the thalamic mediodorsal nucleus in the rat medial prefrontal cortex. Brain Res. (1984) 302:257–65. 10.1016/0006-8993(84)90238-5 6733513

[85] Piray P Zeighami Y Bahrami F Eissa AM Hewedi DH Moustafa AA. Impulse control disorders in Parkinson's disease are associated with dysfunction in stimulus valuation but not action valuation. J Neurosci. (2014) 34:7814–24. 10.1523/JNEUROSCI.4063-13.2014 24899705 PMC6608260

[86] Paulus MP Hozack N Frank L Brown GG. Error rate and outcome predictability affect neural activation in prefrontal cortex and anterior cingulate during decision-making. Neuroimage. (2002) 15:836–46. 10.1006/nimg.2001.1031 11906224

[87] Diederich NJ Goldman JG Stebbins GT Goetz CG. Failing as doorman and disc jockey at the same time: amygdalar dysfunction in Parkinson's disease. Mov Disord. (2016) 31:11–22. 10.1002/mds.26460 26650182

[88] Ambroggi F Ishikawa A Fields HL Nicola SM. Basolateral amygdala neurons facilitate reward-seeking behavior by exciting nucleus accumbens neurons. Neuron. (2008) 59:648–61. 10.1016/j.neuron.2008.07.004 18760700 PMC2603341

[89] Tessitore A Santangelo G De Micco R Vitale C Giordano A Raimo S. Cortical thickness changes in patients with Parkinson's disease and impulse control disorders. Parkinsonism Relat Disord. (2016) 24:119–25. 10.1016/j.parkreldis.2015.10.013 26810913

[90] Yoo HB Lee JY Lee JS Kang H Kim YK Song IC. Whole-brain diffusion-tensor changes in parkinsonian patients with impulse control disorders. J Clin Neurol. (2015) 11:42–7. 10.3988/jcn.2015.11.1.42 25628736 PMC4302178

[91] Petersen K Van Wouwe N Stark A Lin YC Kang H Trujillo-Diaz P. Ventral striatal network connectivity reflects reward learning and behavior in patients with Parkinson's disease. Hum Brain Mapp. (2018) 39:509–21. 10.1002/hbm.23860 29086460 PMC5718974

[92] Munro CA McCaul ME Wong DF Oswald LM Zhou Y Brasic J. Sex differences in striatal dopamine release in healthy adults. Biol Psychiatry. (2006) 59:966–74. 10.1016/j.biopsych.2006.01.008 16616726

[93] Palermo S Morese R Zibetti M Dematteis F Sirgiovanni S Stanziano M. Impulse control disorder and response-inhibition alterations in Parkinson's disease. A rare case of totally absent functionality of the medial-prefrontal cortex and review of literature. J Adv Res. (2017) 8:713–6. 10.1016/j.jare.2017.09.004 29034115 PMC5633757

[94] van Eimeren T Pellecchia G Cilia R Ballanger B Steeves TD Houle S. Drug-induced deactivation of inhibitory networks predicts pathological gambling in PD. Neurology. (2010) 75:1711–6. 10.1212/WNL.0b013e3181fc27fa 20926784 PMC3033606

[95] Seeman P. Parkinson's disease treatment may cause impulse-control disorder via dopamine D3 receptors. Synapse. (2015) 69:183–9. 10.1002/syn.21805 25645960

[96] Leplow B Sepke M Schonfeld R Pohl J Oelsner H Latzko L. Impaired learning of punishments in Parkinson's disease with and without impulse control disorder. J Neural Transm. (2017) 124:217–25. 10.1007/s00702-016-1648-9 27848033

[97] Cools R Lewis SJ Clark L Barker RA Robbins TW. L-DOPA disrupts activity in the nucleus accumbens during reversal learning in Parkinson's disease. Neuropsychopharmacology. (2007) 32:180–9. 10.1038/sj.npp.1301153 16841074

[98] Preuschoff K Bossaerts P Quartz SR. Neural differentiation of expected reward and risk in human subcortical structures. Neuron. (2006) 51:381–90. 10.1016/j.neuron.2006.06.024 16880132

[99] Chambers RA Taylor JR Potenza MN. Developmental neurocircuitry of motivation in adolescence: a critical period of addiction vulnerability. Am J Psychiatry. (2003) 160:1041–1052. 10.1176/appi.ajp.160.6.1041 12777258 PMC2919168

[100] Claassen DO Stark AJ Spears CA Petersen KJ van Wouwe NC Kessler RM. Mesocorticolimbic hemodynamic response in Parkinson's disease patients with compulsive behaviors. Mov Disord. (2017) 32:1574–83. 10.1002/mds.27047 28627133 PMC5681361

[101] O'Doherty J Kringelbach ML Rolls ET Hornak J Andrews C. Abstract reward and punishment representations in the human orbitofrontal cortex. Nat Neurosci. (2001) 4:95–102. 10.1038/82959 11135651

[102] Volkow ND Fowler JS Wang GJ The addicted human brain viewed in the light of imaging studies: brain circuits and treatment strategies. Neuropharmacology. (2004) 47(Suppl. 1):3–13. 10.1016/j.neuropharm.2004.07.019 15464121

[103] Voon V Potenza MN Thomsen T. Medication-related impulse control and repetitive behaviors in Parkinson's disease. Curr Opin Neurol. (2007) 20:484–92. 10.1097/WCO.0b013e32826fbc8f 17620886

[104] Shaw MC Forbush KT Schlinder J Rosenman E Black DW. The effect of pathological gambling on families, marriages, and children. CNS Spectr. (2007) 12:615–22. 10.1017/S1092852900021416 17667890

[105] Roland KP Jenkins ME Johnson AM. An exploration of the burden experienced by spousal caregivers of individuals with Parkinson's disease. Mov Disord. (2010) 25:189–93. 10.1002/mds.22939 20063397

[106] Mamikonyan E Siderowf AD Duda JE Potenza MN Horn S Stern MB. Long-term follow-up of impulse control disorders in Parkinson's disease. Mov Disord. (2008) 23:75–80. 10.1002/mds.21770 17960796 PMC2651355

[107] Rabinak CA Nirenberg MJ. Dopamine agonist withdrawal syndrome in Parkinson disease. Arch Neurol. (2010) 67:58–63. 10.1001/archneurol.2009.294 20065130

[108] Pondal M Marras C Miyasaki J Moro E Armstrong MJ Strafella AP. Clinical features of dopamine agonist withdrawal syndrome in a movement disorders clinic. J Neurol Neurosurg Psychiatry. (2013) 84:130–5. 10.1136/jnnp-2012-302684 22933817

[109] Cunnington AL White L Hood K. Identification of possible risk factors for the development of dopamine agonist withdrawal syndrome in Parkinson's disease. Parkinsonism Relat Disord. (2012) 18:1051–2. 10.1016/j.parkreldis.2012.05.012 22677468

[110] Rossi PJ Shute JB Opri E Molina R Peden C Castellanos O. Impulsivity in Parkinson's disease is associated with altered subthalamic but not globus pallidus internus activity. J Neurol Neurosurg Psychiatry. (2017) 88:968–70. 10.1136/jnnp-2016-315325 28822983

[111] Lhommee E Boyer F Wack M Pelissier P Klinger H Schmitt E. Personality, dopamine, and Parkinson's disease: insights from subthalamic stimulation. Mov Disord. (2017) 32:1191–200. 10.1002/mds.27065 28643887

[112] Lhommee E Klinger H Thobois S Schmitt E Ardouin C Bichon A. Subthalamic stimulation in Parkinson's disease: restoring the balance of motivated behaviours. Brain. (2012) 135:1463–77. 10.1093/brain/aws078 22508959

[113] Adams WK Vonder Haar C Tremblay M Cocker PJ Silveira MM Kaur S. Deep-brain stimulation of the subthalamic nucleus selectively decreases risky choice in risk-preferring rats. eNeuro. (2017) 4:e0094–17. 10.1523/ENEURO.0094-17.2017 28791332 PMC5547195

[114] Merola A Romagnolo A Rizzi L Rizzone MG Zibetti M Lanotte M. Impulse control behaviors and subthalamic deep brain stimulation in Parkinson disease. J Neurol. (2017) 264:40–48. 10.1007/s00415-016-8314-x 27761641

[115] Ardouin C Voon V Worbe Y Abouazar N Czernecki V Hosseini H. Pathological gambling in Parkinson's disease improves on chronic subthalamic nucleus stimulation. Mov Disord. (2006) 21:1941–6. 10.1002/mds.21098 16972268

[116] Castrioto A Funkiewiez A Debu B Cools R Lhommee E Ardouin C. Iowa gambling task impairment in Parkinson's disease can be normalised by reduction of dopaminergic medication after subthalamic stimulation. J Neurol Neurosurg Psychiatry. (2015) 86:186–90. 10.1136/jnnp-2013-307146 24860137

[117] Knobel D Aybek S Pollo C Vingerhoets FJ Berney A. Rapid resolution of dopamine dysregulation syndrome (DDS) after subthalamic DBS for Parkinson disease (PD): a case report. Cogn Behav Neurol. (2008) 21:187–9. 10.1097/WNN.0b013e318185e6e2 18797262

[118] Alberts JL Voelcker-Rehage C Hallahan K Vitek M Bamzai R Vitek JL. Bilateral subthalamic stimulation impairs cognitive-motor performance in Parkinson's disease patients. Brain. (2008) 131:3348–60. 10.1093/brain/awn238 18842609 PMC2639204

[119] Lim SY O'Sullivan SS Kotschet K Gallagher DA Lacey C Lawrence AD. Dopamine dysregulation syndrome, impulse control disorders and punding after deep brain stimulation surgery for Parkinson's disease. J Clin Neurosci. (2009) 16:1148–52. 10.1016/j.jocn.2008.12.010 19553125

[120] Moum SJ Price CC Limotai N Oyama G Ward H Jacobson C. Effects of STN and GPi deep brain stimulation on impulse control disorders and dopamine dysregulation syndrome. PLoS ONE. (2012) 7:e29768. 10.1371/journal.pone.0029768 22295068 PMC3266249

[121] Smeding HM Goudriaan AE Foncke EM Schuurman PR Speelman JD Schmand B. Pathological gambling after bilateral subthalamic nucleus stimulation in Parkinson disease. J Neurol Neurosurg Psychiatry. (2007) 78:517–9. 10.1136/jnnp.2006.102061 17210626 PMC2117849

[122] De la Casa-Fages B Grandas F. Dopamine dysregulation syndrome after deep brain stimulation of the subthalamic nucleus in Parkinson's disease. J Neurol Sci. (2012) 312:191–3. 10.1016/j.jns.2011.08.014 21872889

[123] Le Jeune F Peron J Grandjean D Drapier S Haegelen C Garin E. Subthalamic nucleus stimulation affects limbic and associative circuits: a PET study. Eur J Nucl Med Mol Imaging. (2010) 37:1512–20. 10.1007/s00259-010-1436-y 20349231

[124] Castrioto A Lhommee E Moro E Krack P. Mood and behavioural effects of subthalamic stimulation in Parkinson's disease. Lancet Neurol. (2014) 13:287–305. 10.1016/S1474-4422(13)70294-1 24556007

[125] Thomas A Bonanni L Gambi F Di Iorio A Onofrj M. Pathological gambling in Parkinson disease is reduced by amantadine. Ann Neurol. (2010) 68:400–4. 10.1002/ana.22029 20687121

[126] Kashihara K Imamura T. Amantadine may reverse punding in Parkinson's disease–observation in a patient. Mov Disord. (2008) 23:129–30. 10.1002/mds.21780 17960816

[127] Cera N Bifolchetti S Martinotti G Gambi F Sepede G Onofrj M. Amantadine and cognitive flexibility: decision making in Parkinson's patients with severe pathological gambling and other impulse control disorders. Neuropsychiatr Dis Treat. (2014) 10:1093–101. 10.2147/NDT.S54423 24971012 PMC4069151

[128] Weintraub D Sohr M Potenza MN Siderowf AD Stacy M Voon V. Amantadine use associated with impulse control disorders in Parkinson disease in cross-sectional study. Ann Neurol. (2010) 68:963–8. 10.1002/ana.22164 21154480

[129] Todorova A Samuel M Brown RG Chaudhuri KR. Infusion therapies and development of impulse control disorders in advanced Parkinson disease: clinical experience after 3 years' follow-up. Clin Neuropharmacol. (2015) 38:132–4. 10.1097/WNF.0000000000000091 26166238

[130] Catalan MJ de Pablo-Fernandez E Villanueva C Fernandez-Diez S Lapena-Montero T Garcia-Ramos R. Levodopa infusion improves impulsivity and dopamine dysregulation syndrome in Parkinson's disease. Mov Disord. (2013) 28:2007–10. 10.1002/mds.25636 24123193

[131] Rotondo A Bosco D Plastino M Consoli A Bosco F. Clozapine for medication-related pathological gambling in Parkinson disease. Mov Disord. (2010) 25:1994–5. 10.1002/mds.23177 20669252

[132] Hardwick A Ward H Hassan A Romrell J Okun MS. Clozapine as a potential treatment for refractory impulsive, compulsive, and punding behaviors in Parkinson's disease. Neurocase. (2013) 19:587–91. 10.1080/13554794.2012.713490 22934916

[133] Bonfils NA Benyamina A Aubin HJ Luquiens A. Clozapine use for refractory impulse control disorders in Parkinson's disease: a case report. Psychopharmacology. (2015) 232:3677–9. 10.1007/s00213-015-4048-1 26289354

[134] Nardone R De Blasi P Holler Y Christova M Tezzon F Trinka E. Repetitive transcranial magnetic stimulation transiently reduces punding in Parkinson's disease: a preliminary study. J Neural Transm. (2014) 121:267–74. 10.1007/s00702-013-1100-3 24132699

[135] Okai D Askey-Jones S Samuel M O'Sullivan SS Chaudhuri KR Martin A. Trial of CBT for impulse control behaviors affecting Parkinson patients and their caregivers. Neurology. (2013) 80:792–9. 10.1212/WNL.0b013e3182840678 23325911 PMC3598451

[136] Sriram A Ward HE Hassan A Iyer S Foote KD Rodriguez RL. Valproate as a treatment for dopamine dysregulation syndrome (DDS) in Parkinson's disease. J Neurol. (2013) 260:521–7. 10.1007/s00415-012-6669-1 23007193

[137] Kraus SW Etuk R Potenza MN. Current pharmacotherapy for gambling disorder: a systematic review. Expert Opin Pharmacother. (2020) 21:287–96. 10.1080/14656566.2019.1702969 31928246

